# A spatiotemporal mixed model to assess the influence of environmental and socioeconomic factors on the incidence of hand, foot and mouth disease

**DOI:** 10.1186/s12889-018-5169-3

**Published:** 2018-02-20

**Authors:** Lianfa Li, Wenyang Qiu, Chengdong Xu, Jinfeng Wang

**Affiliations:** 1 LREIS, Institute of Geographical Sciences and Natural Resources Research, Chinese Academy of Sciences, Beijing, China; 2 University of Chinese Academy of Sciences, Beijing, 10049 China

**Keywords:** Spatiotemporal mixed model, Spatial effect, Non-linear effect, Hand-foot-mouth disease, Spatiotemporal scanning statistics

## Abstract

**Background:**

As a common infectious disease, hand, foot and mouth disease (HFMD) is affected by multiple environmental and socioeconomic factors, and its pathogenesis is complex. Furthermore, the transmission of HFMD is characterized by strong spatial clustering and autocorrelation, and the classical statistical approach may be biased without consideration of spatial autocorrelation. In this paper, we propose to embed spatial characteristics into a spatiotemporal additive model to improve HFMD incidence assessment.

**Methods:**

Using incidence data (6439 samples from 137 monitoring district) for Shandong Province, China, along with meteorological, environmental and socioeconomic spatial and spatiotemporal covariate data, we proposed a spatiotemporal mixed model to estimate HFMD incidence. Geo-additive regression was used to model the non-linear effects of the covariates on the incidence risk of HFMD in univariate and multivariate models. Furthermore, the spatial effect was constructed to capture spatial autocorrelation at the sub-regional scale, and clusters (hotspots of high risk) were generated using spatiotemporal scanning statistics as a predictor. Linear and non-linear effects were compared to illustrate the usefulness of non-linear associations. Patterns of spatial effects and clusters were explored to illustrate the variation of the HFMD incidence across geographical sub-regions. To validate our approach, 10-fold cross-validation was conducted.

**Results:**

The results showed that there were significant non-linear associations of the temporal index, spatiotemporal meteorological factors and spatial environmental and socioeconomic factors with HFMD incidence. Furthermore, there were strong spatial autocorrelation and clusters for the HFMD incidence. Spatiotemporal meteorological parameters, the normalized difference vegetation index (NDVI), the temporal index, spatiotemporal clustering and spatial effects played important roles as predictors in the multivariate models. Efron’s cross-validation R^2^ of 0.83 was acquired using our approach. The spatial effect accounted for 23% of the R^2^, and notable patterns of the posterior spatial effect were captured.

**Conclusions:**

We developed a geo-additive mixed spatiotemporal model to assess the influence of meteorological, environmental and socioeconomic factors on HFMD incidence and explored spatiotemporal patterns of such incidence. Our approach achieved a competitive performance in cross-validation and revealed strong spatial patterns for the HFMD incidence rate, illustrating important implications for the epidemiology of HFMD.

**Electronic supplementary material:**

The online version of this article (10.1186/s12889-018-5169-3) contains supplementary material, which is available to authorized users.

## Background

Hand, foot and mouth disease (HFMD) is a common infectious disease that mostly occurs in children younger than 5 years of age. This disease is caused by EV71, CoxA16 and other viruses, and it can lead to symptoms in the hand, mouth or foot, including fever, blisters, and ulcers. It can also cause aseptic meningitis, encephalitis, neurogenic edema and other symptoms in some critically ill patients and may even be life threatening [1]. Therefore, via investigation of the associations of the influencing factors, such as meteorological, geo-environmental and socioeconomic variables, with the incidence of HFMD, the critical risk factors and the difference of risk in regions can be identified and provided for decision-making support of prevention and control measures against this disease.

Existing studies have shown that the incidence of HFMD is related to meteorological, geo-environmental and socioeconomic factors. Meteorological factors, such as air temperature and humidity [2], rainfall [3], and wind speed [4], have important effects on the incidence of HFMD. The short-term El Niño effect was reported to be associated with the incidence and spread of HFMD [5]. Socioeconomic factors, including population density, the number of industrial companies and the ratio of students in a population, were identified as important HFMD risk factors [6]. The gross domestic product (GDP) was found to make a great contribution to the incidence of HFMD [7]. Geographical environmental factors, such as the normalized difference vegetation index (NDVI), population density, land cover types and roadway density, were demonstrated to affect the incidence of HFMD [8, 9].

In terms of modeling methods, geographically weighted regression [10], boosted regression tree analysis [11], the generalized additive model [12], and the Bayesian network [13], among others, were used to investigate the relationship between HFMD and influencing factors. Some existing studies [10, 14, 15] used linear regression to model the relationship between influencing factors and incidence. Others showed significant non-linear relationships [4, 16] between the factors and incidence and the effect [7] of interactions between different factors on the incidence. In addition, many studies primarily focused on a single dimension of time or space and not on a systematic combination of the two. The studies on temporal factors include investigations of the seasonal changes of meteorological parameters [4, 15] or identification of the delay effects of influencing factors [17]. The resulting analyses or the models used in these studies often ignored the influence of differences in the areas or spatial autocorrelation on the disease incidence. Spatial models mostly focused on the investigation of the spatial autocorrelation and clustering of the incidence and ignored the temporal effect. Meanwhile, spatiotemporal studies [3, 18, 19] were based on spatiotemporal scans to detect the clustering of HFMD incidence. This widely used method could discover the pattern of HFMD spatiotemporal propagation, but combining it with other factors, such as meteorological and geo-environmental parameters, in these methods was difficult.

In this study, we propose a mixed spatiotemporal model that evaluates the impact of meteorological, socioeconomic and geo-environmental factors on the incidence of HFMD and predicts the incidence risk in the target area within a certain period of time. This model uses geo-additive regression to establish a non-linear relationship between influencing factors and disease incidence and systematically integrates the spatial and temporal autocorrelation and spatiotemporal clustering factors; the contribution of meteorological, environmental, and land-use patterns, the effect of spatial and temporal autocorrelation, and the hotspot output of disease for a robust prediction of HFMD incidence are illustrated. Using cross-validation, our approach demonstrated the improvement in assessment of the disease risk.

## Methods

### Study region

**Fig. 1:**
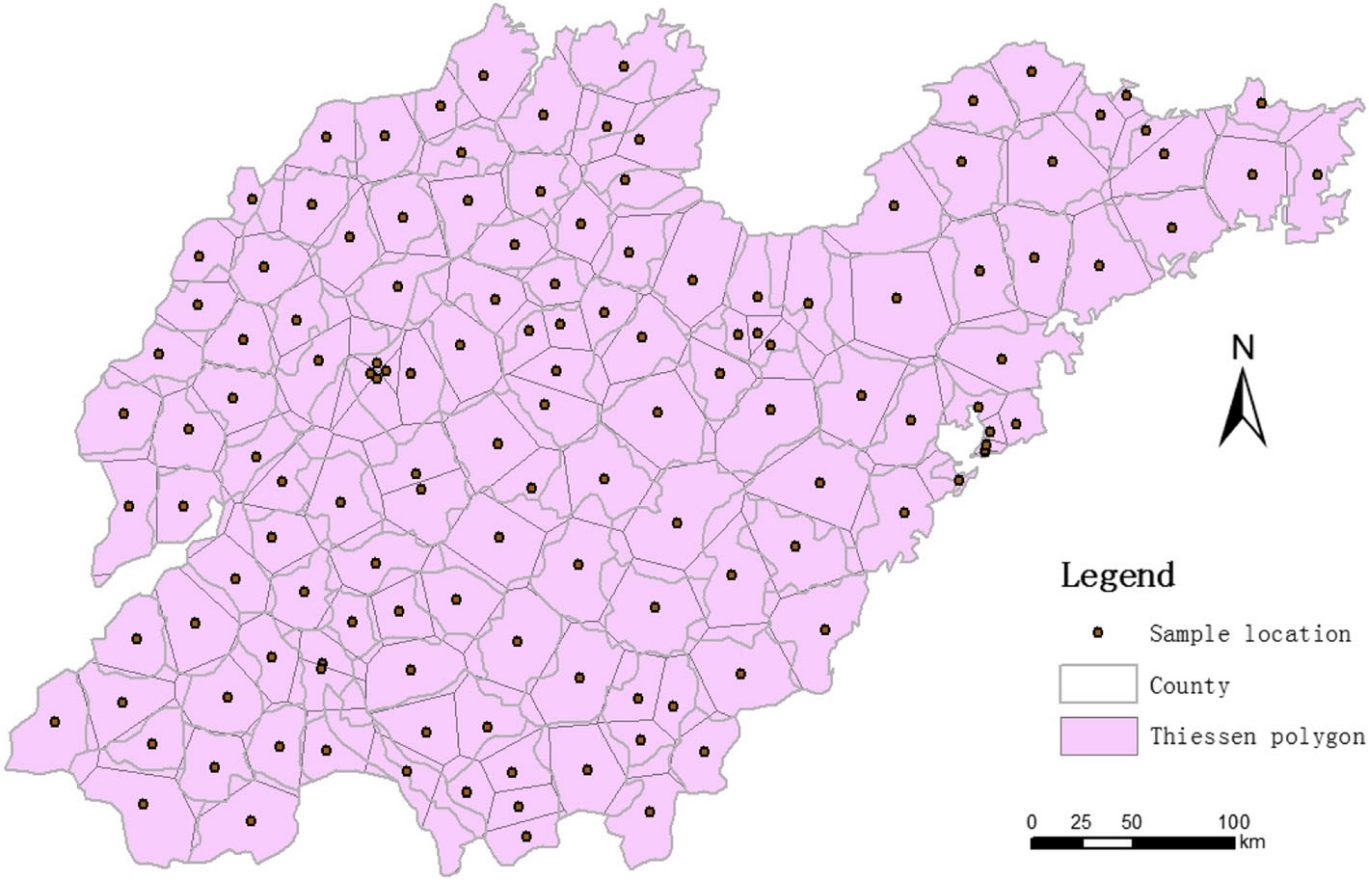
Study region with the sample district’s central location and Thiessen polygons constructed for spatial effects

Shandong Province is located between 34° 25′ and 38° 23′ north latitude and between 114° 36′ and 122° 43′ east longitude (Fig. 1). It is an eastern coastal province of China bordered by the North China Plain to the west. Its eastern part is the Shandong Peninsula, which extends into the Yellow Sea. The total area of Shandong Province is 158,000 km^2^. Shandong has a population of nearly 98 million and is ranked second in China in terms of population. Shandong has a temperate continental monsoon climate with rain and heat in the same quarter. The average annual temperature is 13 degrees Celsius; rainfall is concentrated in the summer, and the annual rainfall is between 550 and 950 mm.

### Incidence rate

From the Chinese Center for Disease Control and Prevention, weekly disease incidence reports for a total of 138 districts in Shandong for 47 weeks beginning May 2008 were obtained for this study. These data were reported by the health departments of the districts. We analyzed the weekly incidence data for each district and county (each district and county is represented by its center point).

### Influencing factors

1. Meteorological station data

Meteorological data were obtained from the China Meteorological Data Network (http://data.cma.cn/en). Data were collected in the same period as the HFMD incidence data and consisted of the observation data of 677 meteorological stations nationwide, including daily average temperature (°C), daily maximum temperature (°C), daily minimum temperature (°C), relative humidity (%), air pressure (hpa) and wind speed (m/s), among others. In the ArcGIS software (ESRI Inc., Redlands, California, the United States), we used the inverse distance weighting method to calculate the weekly average meteorological parameters for each district in Shandong Province.

2) Socioeconomic data

Based on the 2008 statistical yearbook (http://www.stats-sd.gov.cn/), we obtained socioeconomic statistics, including the GDP (Ten Thousand Yuan), the ratio (%) of primary school students in the population and the number of per capita hospital beds, among other statistics. These socioeconomic statistics characterized the HFMD transmission by communication.

3) NDVI data

Derived from the moderate-resolution imaging spectroradiometer (MODIS), the normalized difference vegetation index (NDVI) reflects the coverage of surface vegetation as an environmental indicator for HFMD. We used the MODIS images of four quarters of 2009 with a resolution of 1 km to derive the NDVI. The NDVI values with​​in each district were averaged based on the district’s area and the seasonal intervals to represent this district’s seasonal NDVI.

4) Land cover data

In this study, the 2009 global land cover raster dataset was obtained from GlobCover (http://due.esrin.esa.int/page_globcover.php) with a resolution of 230 m. The study area data were identified and classified into water bodies, man-made areas and natural areas. For each district, we calculated the proportion of each cover type, which was then used as the land cover factor in the model.

5) Road network data

Dense roads typically mean higher population movements, higher economic levels and higher air pollution, whereas a sparse road network density indicates remote distribution of residential locations and underdeveloped economic levels (most likely lower air pollution). We gathered the road network data from OpenStreetMap (http://www.openstreetmap.org/) and calculated the road length (km) of the main roads and secondary roads within each district.

### Spatiotemporal cluster output

This study used the spatiotemporal scanning statistical method [20] to obtain the spatiotemporal clustering data of HFMD. This method used a dynamic cylindrical window in the study area to conduct scanning analysis of the incidence of disease, with the bottom of the cylinder representing the scanned area and the height representing the scanning time length. The difference between the number of disease occurrences in the window and that of the area outside the window is summarized after each change, and the log likelihood ratio is used to test whether the difference is caused by random variation. Windows of statistical significance indicate this region’s clustering trend of the incidence rate, revealing “hotspots” of disease occurrence. In this study, the data of HFMD in 47 districts were calculated using the space-time permutation method [20] in the SaTScan software (https://www.satscan.org). Five hotspots were calculated, and the three most significant hotspots were classified as significant clusters. The other two types of hotspots were classified as sub-significant clusters, and the rest were classified as insignificant clusters.

### Modeling approach

The HFMD incidence is in line with the Poisson distribution, and the modeling frame is constructed using the following formula:

1 $$ \mathit{\log}\left({\mu}_{s,t}\right)= Log\left({P}_s\right)+\log \left({\lambda}_{s,t}\right) $$

2 $$ {\lambda}_{s,t}=\exp \left(\beta 0+{f}_r\left(s,t\right)+{\sum}_{i=1}^ks\Big({x}_i\ \left(s,t\right)\right)+ fac\left({c}_{s,t}\right)+{f}_s\ \left(r(s)\right)+{f}_{re}\left(r\ (s)\right)+\varepsilon $$

where *s* and *t* represent the spatial unit (sub-region) and the time point (weekly), respectively; μ_s*,t*_ represents the number of HFMD cases for region *s* and week *t*, *μ*_*s,t*_~Poisson(*λ*_*s,t*_); *P*_*s*_ represents the population offset to adjust for the difference in the population; *λ*_*s,t*_ is the incidence rate; *β*_0_ represents the average incidence rate for Shandong Province; *x*_*i*_(*s*,*t*) represents spatial or spatiotemporal covariates (meteorological, environmental and socioeconomic); *s*(…) is an additive non-linear function; *c*_*s,t*_ is the output of a spatiotemporal cluster; *fac*(…) is the factor level for the cluster; *f*_*s*_(*r*(*s*)) represents the structured spatial effects; *f*_*re*_(*r*(*s*)) represents the un-structured spatial effects; and *ε* represents the residuals.

We used the first Julian day of each week (*w*) as a non-linear temporal variable in the model to represent the seasonal effect of HFMD incidence across a year.

For the spatial (e.g., socioeconomic factors) or spatiotemporal (meteorological factors) covariates, the non-parametric additive method was used to model the non-linear associations (the Additional file 1).

The spatial effect aims to capture the influence of neighboring regions that was not captured by the available covariates. The spatial effect represents spatial autocorrelation between discrete units such as polygons. In practice, spatial effects may exhibit a strong spatial structure, and they may sometimes be due to the influence of randomness. Thus, the separation of spatial effects between different sources is necessary for modeling [21]. Moreover, when spatial random effects account for spatial autocorrelation not explained by spatial covariates, such effects are specified as structured spatial effects. If structured spatial random effects cannot fully explain spatial correlation, the additive unstructured random effect can be added to the model to reflect the remaining local spatial variation [22]. See the Additional file 2 for details about spatial effect modeling.

We employed restricted penalized maximum likelihood to solve the spatiotemporal mixed-effect model (Eq. (2)). The BayesXsrc and BayesX packages were used to solve the generalized additive model [23, 24] in the R (version 3.3) statistics software. Furthermore, we used the rgdal and spdep packages to generate the Thiessen polygons (Fig. 1).

The contribution of each covariate selected was evaluated in the univariate and multivariate models. Efron’s pseudo R^2^ [25] and CV R^2^ were calculated to measure the count output, and the corresponding residual plot was examined for potential patterns. For validation, we conducted 10-fold cross-validation (CV) (see the Additional file 3 for details).

## Results

### Summary of HFMD cases and correlation with influencing factors

**Fig. 2:**
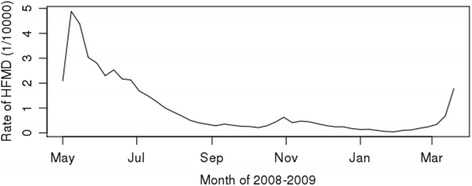
Temporal variation of the incidence from May of 2008 to March of 2009

In total, 6439 weekly samples of HFMD cases were collected from 137 monitoring districts. Each sample included the week index (starting from May 1, 2008, 47 weeks) and the number of cases within the week with the corresponding spatial and spatiotemporal covariates generated. The weekly mean of the HFMD incidence rate was approximately 0.9 per 10,000 population. The mean weekly incidence rate regularly varied with time, and Fig. 2 presents the temporal variation of the mean incidence rate from May 2008 to March 2009 (higher incidence rate in summer and early fall vs. in winter and spring). Table 1 presents the Pearson’s correlation of each covariate with the HFMD incidence rate. The results show a weak correlation ranging from − 0.23 to 0.14, illustrating a possible low estimation performance using linear models for predicting the incidence rate. Thus, non-linearity can better reflect the associations between the influencing factors and the HFMD incidence.

**Table 1:** Correlation and variance explained by the covariate

Covariate	Pearson correlation	Efron’s pseudo R^2^ in univariate model	Contribution to psudo R^2^ in multivariate model
Week id	−0.23	0.10	33.4%
Air pressure	−0.11	0.03	–
Lowest air temperature (°C)	0.14	0.04	0.4%
Higest air temperature (°C)	0.14	–	–
Relative humidity (%)	0.07	0.02	–
Wind speed (m/s)	0.08	0.01	3.1%
Road density	0.15	0.05	–
GDP	0.11	0.03	–
Proportion of primary school students	0.01^a^	0.02	–
Number of hospital beds per capita	0.19	0.06	–
Artificial coverage ratio	0.16	0.15	–
NDVI	−0.04	0.04	4.3%
Spatiotemporal cluster	0.12	0.02	23.0%
Spatial effect		0.22	23.0%
Total pseudo R^2^			87.2%

^a^indicates spatistical insignificance

-indicates no use of the covairate in the multivariate model

### Effects of influencing factors and spatial variation and clusters

**Fig. 3:**
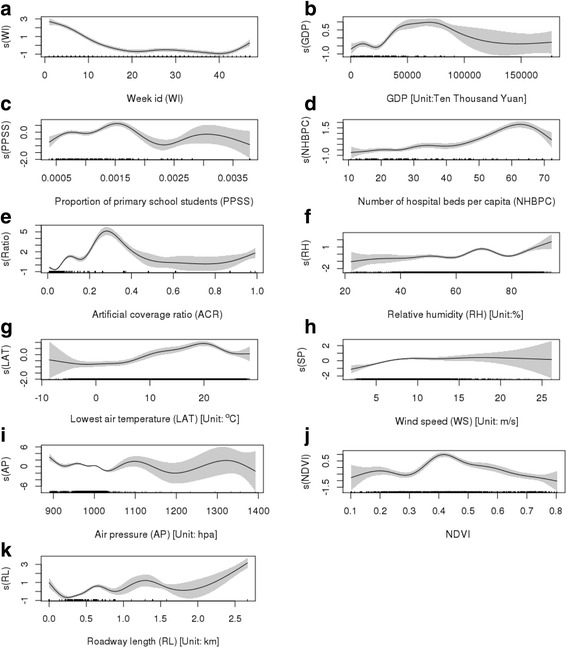
Non-linear association between spatiotemporal covariates [week id (**a**), GDP (**b**), proportion of primary school students (**c**), number of hospital beds per capita (**d**), artificial coverage ratio (**e**), relative humidity (**f**), lowest air temperature (**g**), wind speed (**h**), air pressure (**i**), NDVI (**j**) and roadway length (**k**)] and incidence of HFMD in the univariate models (shade area indicating 95% confidence intervals)

**Fig. 4:**
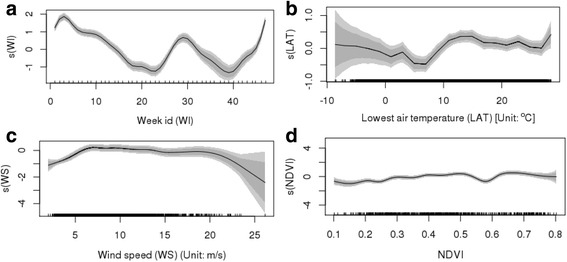
Non-linear association between spatiotemporal covariates [week id (**a**), lowest air temperature (**b**), wind speed (**c**) and NDVI (**d**)] and incidence of HFMD in the multivariate models (shade area indicating 95% confidence intervals)

**Fig. 5:**
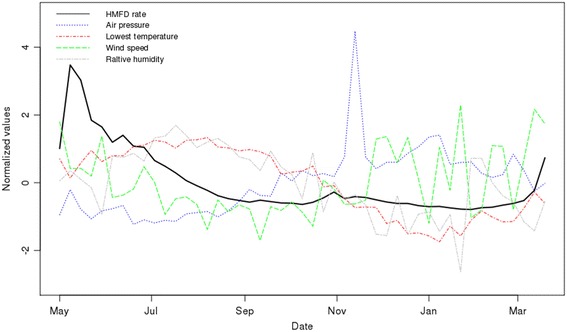
Temporal variations of spatiotemporal covariates (mainly meteorological variables) with HFMD rate

The complex non-linear associations between influencing factors and the HFMD incidence rate were explored in univariate models (Fig. 3) and multivariate models (Fig. 4). Table 1 shows the Pearson’s correlation between the influencing factors and the HFMD incidence rate, the pseudo R^2^ in the univariate models, and each factor’s contribution in the multivariate models. Figure 5 shows the trend of the mean incidence with those of spatiotemporal meteorological factors to illustrate their association.

#### Meteorological factors

The Pearson’s correlation results and non-linear associations in the univariate models showed that the HFMD incidence generally increased with an increase (Fig. 3-f, g, h) in relative humidity and air temperature, and that the incidence decreased with an increase in air pressure (Fig. 3-i). Figure 5 shows that the temporal trends of the weekly mean air temperature, relative humidity and wind speed were basically similar to that of the HFMD incidence rate, and the temporal trend of air pressure was opposite the rate’s trend.

Non-linearity (Fig. 3) captured the complex associations that varied with different value ranges of the meteorological factors and better captured the observed association. The sensitivity test showed that non-linearity improved the model’s performance by 14% in Efron’s R^2^ (0.73 in the linear model vs. 0.87 in the additive model). Although each meteorological factor showed a statistically significant complex non-linear association with the HFMD rate, only two factors (lowest air temperature and wind speed) were selected in the multivariate model (Fig. 5), accounting for 3.5% of the variance (Table 1).

The result (Fig. 3-g) shows that the minimum daily temperature and the risk of disease presented a locally positive association (slope > 0) for the value range smaller than 20 °C for the temperature. The wind speed was shown to be generally positively correlated with HFMD incidence. In addition, as seen in Fig. 3-h, the effect of wind speed on HFMD was found to be locally weakened and even inversed when wind speed was high.

On average, the atmospheric pressure was negatively associated with HFMD incidence; a lower level of pressure might weaken the human immune system’s strength, thus increasing the vulnerability to HFMD [26].

#### Green space

As an environmental factor, the NDVI indicates the green space. In total, the NDVI presented a weak linear association with the incidence, but its non-linear association is complicated (first locally positive and then negative associations), as shown in Fig. 3-j. In the multivariate model, the NDVI explained 4.3% of the variance.

#### Socioeconomic factors

The socioeconomic factors include GDP, the proportion of primary school students and the number of hospital beds per capita, which are spatial covariates without temporal variation. Except for the proportion of primary school students, the socioeconomic covariates were significantly positively associated with the HFMD incidence. In particular, the number of hospital beds per capita accounted for 6% of the variance in the univariate model, illustrating its important influence on the incidence. In the multivariate model, no socioeconomic factors were selected as predictors.

#### Land use and traffic factor

As the land use indicator, the artificial coverage ratio was extracted to reflect the land use pattern, accounting for 15% of the variance in the univariate models. A roadway length within the buffering distance of 5 km indirectly indicated the traffic volume and related air pollution. It is observed to be significantly positively associated with the HFMD incidence rate. The results of the univariate model (Fig. 3-k) presented a general positive association with the HFMD risk.

#### Temporal effect and spatiotemporal clusters

**Fig. 6:**
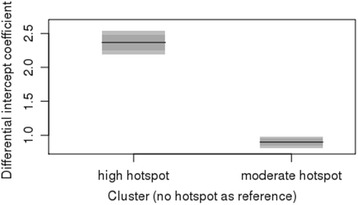
Differential intercept coefficients for spatiotemporal clusters (no cluster as reference factor)

As shown in Fig. 2, the incidence presents a strong temporal pattern, with a high incidence in summer and a low incidence in winter (basically in line with the influence of air temperature). The temporal indicator (weekly index) accounted for 10% of the variance in the univariate model and 33.4% in the multivariate model. The scanning statistics output also presented strong spatiotemporal patterns. The clusters were used as the factor variable in the model (accounting for 23% of the variance), and the results (Fig. 6) showed differential intercept coefficients for the moderate and high hotspots, naturally illustrating a much higher risk for the highest cluster hotspot.

#### Spatial effect

**Fig. 7:**
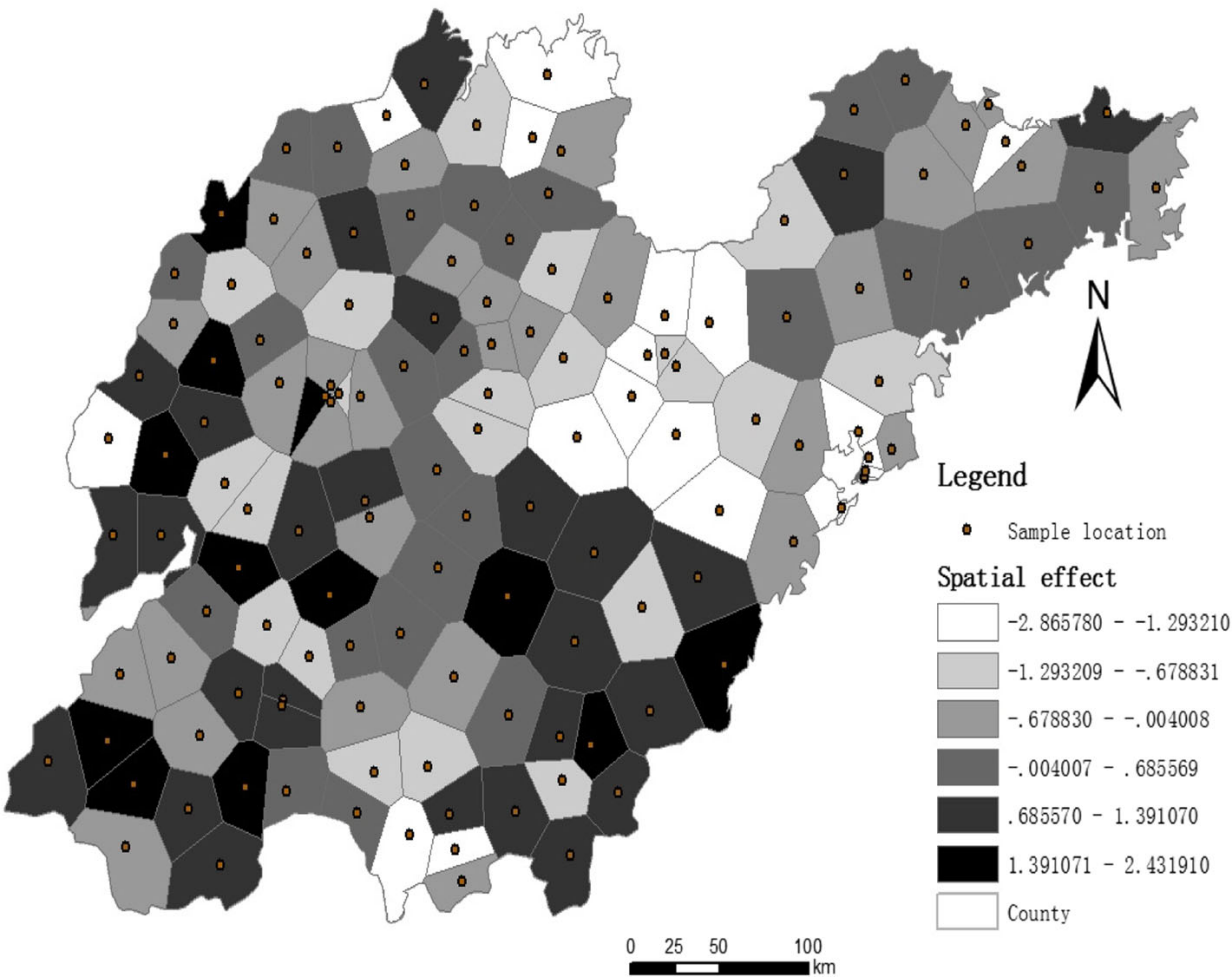
Spatial effects across Thiessen polygons

The spatial effect played an important role, explaining 23% of the variance in the multivariate model (Table 1). Figure 7 presented spatially distributed patterns of spatial effect in the results.

### Validation

**Fig. 8:**
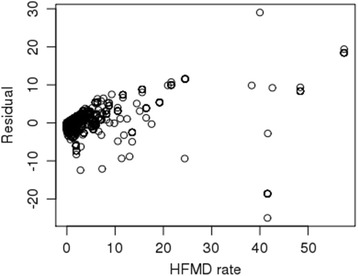
Residual plot for the observed HFMD rate

Efron’s R^2^ is 0.87, with a CV R^2^ of 0.83 in the 10-fold cross-validation. Only six predictors were selected as the predictors in the multivariate model. The six predictors included two spatiotemporal meteorological covariates (lowest air temperature and wind speed), the NDVI, the spatial effect, the spatiotemporal cluster output and the weekly index. The results illustrate the strong spatiotemporal characteristics of the distribution of the HFMD incidence rate. Figure 8 shows no notable pattern in the residual plot of the observed HFMD incidence rate, illustrating the major variation captured by our approach.

## Discussion

As a gastrointestinal infectious disease, HFMD is caused by the virus of the enterovirus genus (group) that exhibits strong transitivity. HFMD is mainly transmitted by nasopharyngeal secretions such as saliva or nasal mucus, by direct contact, or by fecal-oral transmission. Its latent infection is high and can cause large epidemics in short periods of time [27]. The transmission paths are complex and affected by multiple factors. We studied the complex associations between influencing factors and the HFMD incidence rate using the non-linear modeling approach with embedding of spatial effect.

Meteorological factors have significant influences on the HFMD incidence rate [28]. Our results are consistent with previous studies on the influence of air temperature [2] and relative humidity [2, 12]. The exact mechanism related to the association of meteorological parameters with HFMD incidence is not very clear. It is generally assumed that meteorological parameters affect HFMD transmission and then its incidence rate. Several factors, such as pathogen infectivity, human behavior patterns, and immune system fluctuations, were proposed to account for such associations [29].

Our results provide the informative non-linear associations of the influencing factors with HFMD risk and such non-linear association consists of multiple linear associations corresponding to different value ranges of the covariate in the additive models (the Additional file 1). Previous studies identified a non-linear association between relative humidity and the risk of HFMD. Zhang et al. [4] found extreme values ​​of 45% (minima) and 85% (maxima), and the change between the extreme values indicated a linear association, with the risk of disease occurrence increasing with increased humidity. This view was also supported in the studies by Chen et al. [12] and Nguyen et al. [17]. A possible explanation for this finding is that the enterovirus’ survival is generally assumed to be proportional to a low-moderate relative humidity level between the temperatures of 20 °C and 33 °C [30] and favored by higher moderate humidity because the virus can persist longer on inanimate surfaces [31]. Furthermore, our study found an extremum near 70%, and an increase in relative humidity in the range of 70% to 80% led to decreased disease occurrence, which is similar to the results of Urashima et al. [32]. The period from September to November is associated with relatively high humidity and is the season with a low incidence of HFMD (Fig. 5); there is a negative correlation between relative humidity and incidence in this season. Liao et al. [15] suggested that the virus was inhibited after the increase in relative humidity reached a threshold, thereby reducing the risk of disease.

For the non-linear association between the minimum daily temperature and HFMD incidence, there are two potential reasons: (1) virological evidence shows the temperature-sensitive nature of enteroviruses and other human enteric viruses [33, 34], and (2) more outdoor activities in moderately warmer weather increase close contact between individuals, thus enhancing the HFMD transmission. Furthermore, our result in the univariate model identified 20 °C as a critical point beyond which the minimum temperature and the risk of HFMD disease showed a locally negative association (slope < 0) for the value range beyond 20 °C. The study by Xu et al. [16] in Beijing and that by Huang et al. [35] in Guangzhou also found similar patterns. This inverse association between HFMD and higher air temperature is unclear. We speculate that children are more likely to stay indoors during the season with higher daily minimum temperatures, reducing the impact from public places and crowds. In addition, our study was conducted at the spatiotemporal level, and the spatial difference in HFMD incidence during the high temperature season might also be a related factor. Due to influence of the other covariates, the multivariate model presented a similar variation of associations with a different threshold (about 16 °C).

Our result about the general association between wind speed with HFMD incidence is similar to the results of [15, 36]. In addition, such an association might locally weakened and even inversed at high wind speed. Although an increase in wind speed is beneficial to the spread of the virus in the air through airborne droplets [37], the virus may only be able to stay in the air for a short period of time under a high wind speed. Outdoor activities are also curtailed in windy weather, which reduces the chance of exposure to the virus.

The association between the NDVI and HFMD risk was rarely investigated in the previous studies. Cao et al. [8] and Stanaway [9] concluded that there was a negative correlation between them. Studies found that urban areas have a high risk of disease, which can be explained by the fact that the vegetation cover is lower in urban areas than in areas with poor economic development and covered by mountains and cultivated land. This study found a non-linear relationship between the two. Compared to our study, the two abovementioned studies were conducted on a spatial scale, thus ignoring the changes in the NDVI over time. We believe that the NDVI increases in the spring and summer, consistent with the HFMD season, thus reflecting a certain upward trend. This phenomenon is more pronounced in urban areas where the NDVI is relatively low.

For most of the socioeconomic factors, they were significantly positively associated with the HFMD incidence. To the best of our knowledge, only a few studies have combined socioeconomic factors with the other factors to assess their effects on the HFMD incidence. Bo et al. [6] found that the number of industrial enterprises and the proportion of students in the population were associated with the incidence of HFMD. Huang et al. [7] concluded that GDP had a significant impact on the risk of HFMD incidence. Zeng et al. [38] suggested that the increase in migratory workers from rural areas to cities was an important risk factor for the occurrence of HFMD. Cao et al. [8] concluded that urban areas had a higher risk of HFMD compared with poor areas, which is similar to our result that GDP and HFMD incidence were positively correlated. In urban areas, the higher population density leads to the easy spread of the virus. A more complete health system in developed areas enables the timely and detailed report of disease data to the higher health sector, leading to a bias toward a higher incidence. In this study, the association between the number of hospital beds per capita and the disease incidence also supports this claim. The results of this study revealed that the proportion of primary school students and the HFMD incidence showed a complex non-linear relationship, indicating that there were many confounding factors affecting their association.

The traffic indicator was not selected in the multivariate model, but the univariate analysis showed that traffic factors, as a factor of air pollution, reflected the possibility that air pollution might lead to an increased risk of HFMD. It is generally believed that an increase in particulate matter in the air makes it easier for the virus to attach to particular matter, thus contributing to the spread of the virus [5]. Air pollution can also reduce human immunity and increase the risk in the exposed population [6].

Besides the physical factors, the temporal indicator (weekly index) also played the important role. The result of scanning statistics showed much higher risk for the highest cluster hotspot.

The spatial effect presents the spatial distribution of the HFMD incidence rate across the study region. The posterior spatial effect showed a general increased risk of HFMD in the southwest part of Shandong Province, in contrast with the decreased risk of incidence in the central north and northeast parts of Shandong Province. The HFMD transmission was complicated and closely associated with the population density and communication, which presented strong spatial patterns [39]. Whereas the HFMD transmission cannot be fully captured by the covariates used, the spatial effect and clusters embedded in the models could capture such a spatial pattern (strong spatial autocorrelation), thus considerably improving the model’s performance. Furthermore, the introduction of spatiotemporal scanning statistics and spatial effects accounts for most of the variability caused by the other factors, thus lowering the contributions of the other factors, such as meteorological and traffic factors, in the model. The results showed the important implications of strong spatiotemporal and spatial patterns for HFMD risk assessment.

To the best of our knowledge, this study is one of the first studies to design a geo-additive model to estimate the HFMD incidence rate. We performed a comprehensive exploration of the influence of environmental, meteorological, land use and socioeconomic factors on the HFMD incidence rate in terms of non-linear and spatial effects. Our approach incorporated spatial effects as an indicator of spatial autocorrelation and spatiotemporal cluster output within the model. Due to strong spatiotemporal patterns in the variation of the HFMD incidence, the multivariate model achieved good estimation accuracy (CV R^2^ of 0.83). Our exploration of the influence of various factors and spatiotemporal patterns has important implications for the assessment of the HFMD incidence, and our model provides a good estimation of the HFMD risk, which is useful for decision-making support for HFMD zonation and warning.

This study has several limitations. First, the socioeconomic factor data used in this study do not contain temporal changes. However, socioeconomic factors such as GDP and the number of students do not have significant changes over time within one year. Therefore, the above limitation has a very limited impact on the results. Second, we chose the Thiessen polygon for spatial effect modeling. The Thiessen polygon is affected by the distribution of sample points. However, if more data become subsequently available, the Thiessen polygons can be updated to produce results with better spatial resolution. Third, we chose many variables that might lead to over-fitting in the non-linear additive model. However, the final multivariate model selected fewer variables, strong temporal and spatial variability explained more variation, and cross-validation demonstrated the prediction efficacy of this method. Fourth, the spatial effect and spatiotemporal clustering of the final model explained a large portion of the variation, and the physical meaning of other variables was ignored. However, this study already explored the effects of individual factors on HFMD itself. In terms of prediction accuracy, the contribution of environmental and socioeconomic factors alone was limited. The addition of spatial autocorrelation and spatiotemporal clustering greatly improved the prediction performance. Under the conditions that the influencing factors were complex and the variability of the disease incidence could not be captured effectively, the addition of spatial autocorrelation and spatial clustering items to the model improved the accuracy of risk identification, which was helpful for zoning and warning of HFMD. Last, our model was trained using the data for Shandong Province of China, and the model was applied only for the assessment of the HFMD risk in Shandong Province of China. However, our geo-additive approach, as an improvement of our previous approach [40], can be easily extended to other regions and other infectious diseases similar to HFMD that characterize strong spatial autocorrelation and temporal patterns.

## Conclusions

In this study, a spatiotemporal geo-additive model was designed to analyze the non-linear associations between predictive factors (meteorological, socioeconomic and geo-environmental variables) and the incidence of HFMD. This model incorporated spatiotemporal clustering predictor and spatial autocorrelation effects to characterize spatiotemporal patterns of HFMD incidence. The results presented non-linear associations between the meteorological, land-use, NDVI and socioeconomic factors and the HFMD incidence and revealed notable spatiotemporal patterns of the distribution of the HFMD risk. Cross-validation demonstrated the robust performance of our approach. The results showed the implication for prevention and control of HFMD, and our approach can also be applied to other regions for risk assessment of infectious diseases such as HFMD.

## Additional files

Additional file 1: Appendix 1. Non-linear modeling. (DOCX 14 kb)

Additional file 2: Appendix 2. Spatial effect modeling. (DOCX 18 kb)

Additional file 3: Appendix 3. Cross-validation. (DOCX 15 kb)

## Abbreviations

CDC: Chinese Center for Disease Control and Prevention
CV: Cross-validation
GAM: Generalized additive model
GDP: Gross domestic product
GWR: Geographically weighted regression
HFMD: Hand, foot and mouth disease
NDVI: Normalized difference vegetation index
SVD: Singular value decomposition
