# Supplementary material for: A spatiotemporal mixed model to assess the influence of environmental and socioeconomic factors on the incidence of hand, foot and mouth disease
Source: BMC Public Health. 2018 Feb 20;18:274. doi: 10.1186/s12889-018-5169-3 (PMC5819665; doi:10.1186/s12889-018-5169-3)
Supplement: Supplementary file 1 — Appendix 1. Non-linear modeling. (DOCX 14 kb) [file 12889_2018_5169_MOESM1_ESM.docx]

**Appendix 1. Non-linear modeling**

The following equation illustrates the non-parametric function form used to quantify the non-linear association, *s*(*…*), i.e., approximated by the weighted sum of polynomial spline (B-spline basis) functions:

$x_{min}=\zeta_{0}<\zeta_{1}\ldots<\zeta_{m-1}<\zeta_{m}=<x_{max}$ (1)

$f\left( x_{i} \right)=\sum_{j} \beta_{ij}B_{ij}(x_{i})$ (2)

where *ζ_i_* is the split for the intervals of the covariate *x_i_* (*ζ*_0_ and *ζ_m_* are the minimum and maximum values of *x_i_*, respectively) and *Β_ij_* and *β_ij_* represent the basis function and parameters for the interval *j* for covariate *x_i_*, respectively. In practice, penalty functions were constructed to ensure smoothness between the parameters of adjacent basis functions and to avoid over-fitting.
