# Supplementary material for: A spatiotemporal mixed model to assess the influence of environmental and socioeconomic factors on the incidence of hand, foot and mouth disease
Source: BMC Public Health. 2018 Feb 20;18:274. doi: 10.1186/s12889-018-5169-3 (PMC5819665; doi:10.1186/s12889-018-5169-3)
Supplement: Supplementary file 2 — Appendix 2. Spatial effect modeling. (DOCX 18 kb) [file 12889_2018_5169_MOESM2_ESM.docx]

**Appendix 2. Spatial effect modeling**

In our model, the spatial effects were modeled using the following formulas for spatially structured (5) and spatially unstructured effects (6):

$f_{s}\left( r\left( s \right) \right)=\gamma_{r},r^{'}\neq r\sim N(\frac{1}{N_{r}}\sum_{r^{'}\in\delta_{r}} \gamma_{r},\frac{\tau^{2}}{N_{r}}$ (1)

$f_{re}\left( r\left( s \right) \right)=\gamma_{re}\sim N(0,\tau^{2})$ (2)

where *r*(*s*) is the region in which the observed incidence rate *y*(*s*,*t*) is located, *δ_r_* represents a set of neighbors of region *r*, *N_r_* is the number of neighboring regions for *r*, and *τ*^2^ is the variance component, *τ*^2^ ~IG(*a*,*b*). In (1), *γ_r_* represents the spatial effect of the HFMD incidence rate from neighboring polygons; in (2), *γ_re_* represents the unstructured spatial effect with zero mean and standard deviation (*τ*).

To better model spatial effects, Thiessen polygons were constructed around each sample location (the central location of each district) of HFMD. Thiessen polygons are often used to determine the density of point samples and to build meshes for space-discretized simulation. A uniform distribution of triangles can be used, with avoidance of triangles that are too narrow and irregular [1]. In our model, the spatial effect was used as a random variable of the Thiessen polygon constructed and included formally as a nonparametric additive term in the model.

**References**

1. Seidel R: **The upper bound theorem for polytopes: an easy proof of its asymptotic version**. *Computational Geometry* 1995, **5**(2):115–116.
