# Supplementary material for: A spatiotemporal mixed model to assess the influence of environmental and socioeconomic factors on the incidence of hand, foot and mouth disease
Source: BMC Public Health. 2018 Feb 20;18:274. doi: 10.1186/s12889-018-5169-3 (PMC5819665; doi:10.1186/s12889-018-5169-3)
Supplement: Supplementary file 3 — Appendix 3. Cross-validation. (DOCX 15 kb) [file 12889_2018_5169_MOESM3_ESM.docx]

**Appendix 3. Cross-validation**

In this paper’s 10-fold CV, the original data were randomly partitioned into 10 equal-size subsets. Of the 10 subsets, a single subset was retained as the validation data for testing the model, and the remaining 9 subset were used to train the model. This process was then repeated 10 times, with each of the 10 subsets used exactly once as the validation data. The test results from the 10 folds were then combined to obtain a single estimation from which the performance metrics (R^2^) were computed.
